# Characterizing modulated structures with first-principles calculations: a unified superspace scheme of ordering in mullite

**DOI:** 10.1107/S2053273319000846

**Published:** 2019-02-12

**Authors:** Paul Benjamin Klar, Iñigo Etxebarria, Gotzon Madariaga

**Affiliations:** a Departamento de Física de la Materia Condensada, Facultad de Ciencia y Tecnología, Universidad del País Vasco UPV/EHU, Apartado 644, Bilbao, 48080, Spain; b Departamento de Física Aplicada II, Facultad de Ciencia y Tecnología, Universidad del País Vasco UPV/EHU, Apartado 644, Bilbao, 48080, Spain

**Keywords:** density functional theory (DFT), superspace, modulation functions, Al/Si ordering, silicates, mullite

## Abstract

The benefit of computational methods applying density functional theory for the description and understanding of modulated crystal structures is investigated. A method is presented which allows one to establish, improve and test superspace models including displacive and occupational modulation functions from first-principles calculations on commensurate structures. The total energies of different configurations allow one to distinguish stable and less stable structure models. The study is based on a series of geometrically optimized superstructures of mullite (Al_4+2*x*_Si_2−2*x*_O_10−*x*_) derived from the superspace group *Pbam*(α0½)0*ss*. Despite the disordered and structurally complex nature of mullite, the calculations on ordered superstructures are very useful for determining the ideal Al/Si ordering in mullite, extracting atomic modulation functions as well as understanding the SiO_2_–Al_2_O_3_ phase diagram. The results are compared with experimentally established models which confirm the validity and utility of the presented method.

## Abstract 2

Quantitative parameters of modulated crystal structures are determined from density functional theory calculations applied to superstructures obtained using the superspace formalism. The example of mullite is used to demonstrate that with this approach versatile aspects ranging from ordering phenomena to phase diagrams can be understood on a new level.

## 1. Introduction   

Computational methods have become a valuable tool in crystallography, partly triggered by the steadily improving computer power. Examples include relativistic Hartree–Fock calculations of atomic form factors as listed in the *Inter­national Tables for X-ray Crystallography* (Doyle & Turner, 1968 ▸), the estimation of anisotropic displacement parameters (ADPs) from phonon calculations (Madsen *et al.*, 2013 ▸) or the investigation of phase diagrams (Wang *et al.*, 2005 ▸). All computational methods applied to crystal structure models require invariably fully occupied sites because fractional atoms lack a physical basis. If there are partially occupied atomic sites in the average structure, large defect structures can be generated with random or systematic approaches (Proffen & Neder, 1997 ▸; Okhotnikov *et al.*, 2016 ▸) for further investigation including the simulation of diffuse scattering (Proffen & Neder, 1997 ▸) or molecular dynamics (Matsui, 1996 ▸; Lacks *et al.*, 2005 ▸; Adabifiroozjaei *et al.*, 2018 ▸). For example, to study the structure of highly disordered meta-kaolin an 18-fold supercell with 282 atoms was used for calculations applying density functional theory (DFT) to support the refinement of the pair distribution function (White *et al.*, 2010 ▸). The size of those systems quickly exceeds the possibilities of current *ab initio* calculations on modern clusters, although algorithms were developed to increase the number of atoms per unit cell to a few thousand (Goedecker, 1999 ▸; Mohr *et al.*, 2018 ▸). For even larger systems, force-field (FF) methods are the only choice as these are significantly less demanding in terms of computational cost, but the accuracy is limited and depends strongly on the reliability of the FF parameters with which the atomic and molecular interactions are modelled (Leach, 2001 ▸). Nowadays, DFT methods have become the standard tool to study the structural and electronic properties of crystals when accuracy is essential (Sholl & Steckel, 2009 ▸). First-principles calculations have been applied successfully to a wide range of crystals: from systematic searches of potentially stable phases in multiferroic materials (Diéguez *et al.*, 2011 ▸) to the validation of the structures of molecular crystals (van de Streek & Neumann, 2014 ▸).

Modulated structures are ubiquitous among a great variety of materials like metallic alloys, inorganic, organic and metal–organic compounds, ceramics or minerals (Arakcheeva & Chapuis, 2006 ▸; Bindi, 2008 ▸; Pinheiro & Abakumov, 2015 ▸). Although ordered, the lack of periodicity or the presence of very long spatial periods convert them into systems that require a large number of structural parameters for their description. In the case of strictly aperiodic materials this number becomes infinite. The superspace formalism alleviates this shortcoming, ordering hierarchically by importance the structural details through a moderate and relevant set of symmetry-independent atomic parameters (Van Smaalen, 2004 ▸). Hence, superspace symmetry reveals itself as the most powerful tool for solving, refining and describing modulated structures. Nevertheless, the atomic interactions on which the first-principles calculations are based have not yet been formulated in superspace and initial ideas on ‘superspace DFT’ were not successful (Perez-Mato, 2018 ▸). Therefore, a lot of valuable structural information could remain hidden when first-principles calculations are applied to these kind of materials.

In this study we combine the superspace approach with first-principles calculations to establish superspace models from commensurate superstructures which increases significantly the amount of structural information. The analysis of the energies of the supercells of different compositions allows us to study ordering phenomena and characteristics of phase diagrams in which modulated structures are present.

The method is presented using the example of mullite (Al_4+2*x*_Si_2−2*x*_O_10−*x*_) in the range 0 ≤ *x* ≤ 0.5. Geometrically optimized structures are studied in terms of superspace structural parameters, crystal chemical parameters, lattice parameters, total free energy and respective indications for the phase diagram of the system SiO_2_–Al_2_O_3_. The results are compared with X-ray diffraction (XRD) experiments. As there are several fundamental questions concerning the crystal structure and the phase diagram of mullite, it is an ideal playground to study the capabilities and benefits of first-principles calculations on modulated structures.

### 1.1. Previous DFT studies on modulated structures   

DFT studies on modulated structures are rare and were mostly carried out for commensurate cases or commensurate approximations (Perez-Mato *et al.*, 2007 ▸; Schmidt *et al.*, 2007 ▸; Fredrickson *et al.*, 2008 ▸; Vosswinkel *et al.*, 2018 ▸). A few examples will be mentioned in more detail here.

Fredrickson & Fredrickson (2013 ▸) studied the electronic structure of incommensurately modulated Co_3_Al_4_Si_2_ taking a fivefold supercell and ‘the Al/Si ratios of the mixed sites were rounded to the nearest integer’ to achieve site occupancies that are either 0 or 1. The electronic structure was used to investigate the chemical bonds of the Co atoms. Kilduff & Fredrickson (2016 ▸) observed an incommensurate modulation in CaPd_5+*x*_ compounds and extended the crystal chemical analysis with DFT calculations of a commensurate approximation with composition CaPd_5_. In the cases mentioned the simplest possible input structure was used, which is very advantageous with respect to computational cost. Gulec *et al.* (2016 ▸) observed satellite reflections indicating an incommensurate modulation in Mo_3_Si, but all DFT calculations with up to 30 atoms in the unit cell (*Z* = 7 for Mo_3.28_Si) were based on randomly generated structure models. Zhao *et al.* (2014 ▸) used an adaptive genetic algorithm to find stable crystal structures of ZrCo_5+*x*_. In one compound a structural modulation is clearly detected and mentioned, but not considered for further structural investigation. Izaola *et al.* (2007 ▸) developed a unified superspace model for pyrrhotite (Fe_1−*x*_S) and compared the refined displacive modulation parameters with geometrically optimized superstructures determined with DFT calculations. Apart from the latter study from 2007, we do not know of any investigation that used the superspace formalism in combination with DFT calculations.

### 1.2. Introduction to the mullite system with composition Al_4+2*x*_Si_2−2*x*_O_10−*x*_   

**Figure 1:**
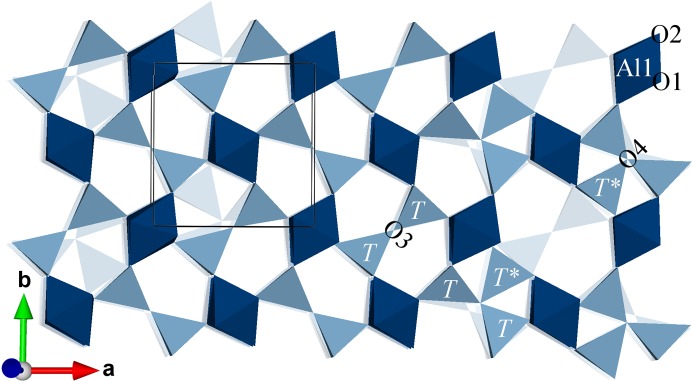
Structural elements of mullite and site labels. Subsequent layers are shown with decreased opacity. Chains of AlO_6_ octahedra (dark blue) are interlinked by diclusters consisting of two *T* sites and triclusters consisting of two *T* sites and a T* site. Two triclusters always embrace the void of an oxygen vacancy like the one between the two labelled T* sites. Octahedrally coordinated Al1 atoms are bonded to four O1 atoms and two O2 atoms. The bridging oxygens of diclusters and triclusters are labelled O3 and O4, respectively. Cations on the *T* site are labelled Al2 or Si2, and on the T* site Al3 or Si3. The unit-cell borders of the mullite average structure are marked by black lines.

At first sight, mullite seems to be the opposite of the ideal candidate for computational studies: the average structure model contains two split-site positions with four partially occupied sites (Sadanaga *et al.*, 1962 ▸; Burnham, 1963 ▸). The real structure can be understood by the presence of oxygen vacancies which cause significant changes to the surrounding tetrahedral environment (Fig. 1 ▸). The concentration of oxygen vacancies *x* is determined by the Al/Si ratio on the tetrahedral sites and may range from 0 to 1, but most mullite samples grown by solid-state reactions and from the melt exhibit a vacancy concentration between *x* ≃ 0.25 and *x* ≃ 0.4 (Schneider *et al.*, 2015 ▸). The precise positions and intensities of satellite reflections with a modulation wavevector **q** = (α 0 ½) depend on the chemical composition as the structural modulation changes with the Al/Si ratio (Agrell & Smith, 1960 ▸; Cameron, 1977*b* ▸; Ylä-Jääski & Nissen, 1983 ▸). Different degrees of ordering exist, but the tendency to long-range vacancy ordering is rather low in mullite (Klar *et al.*, 2018 ▸). For most samples only weak, low-order satellite reflections are visible in reciprocal space and diffuse scattering is present in all reciprocal-space sections due to correlated disorder (Tokonami *et al.*, 1980 ▸; Welberry & Butler, 1996 ▸; Freimann & Rahman, 2001 ▸). The complexity of mullite in reciprocal space must originate from vacancy and Al/Si ordering because sillimanite (Al_2_SiO_5_), which has a very similar average structure, is fully ordered and not modulated (Smith & McConnell, 1966 ▸). Despite several nuclear magnetic resonance measurements and neutron diffraction studies, a clear Al/Si ordering pattern cannot yet be identified (Angel *et al.*, 1991 ▸; Schmücker *et al.*, 2005 ▸; Birkenstock *et al.*, 2015 ▸; Schneider *et al.*, 2015 ▸). From the modulation of the volumes of tetrahedra it was suggested that Si–Si diclusters tend to be sandwiched between vacancies (Klar *et al.*, 2018 ▸).

As the microstructure and composition depend strongly on the experimental conditions (Kriven & Pask, 1983 ▸), different phase diagrams of the system SiO_2_–Al_2_O_3_ emerged over the decades (Aramaki & Roy, 1959 ▸; Aksay & Pask, 1974 ▸; Klug *et al.*, 1987 ▸). A comprehensive review discussed that the 1974 and 1987 phase diagrams, though exhibiting clear differences, are both correct with reference to the different experimental approaches (Aksay *et al.*, 1991 ▸). The characteristics of the phase diagram, especially the borders of the stable solid solution range, could never be explained on a fundamental level.

Two studies on mullite applied DFT to investigate the crystal structure and physical properties. Chen *et al.* (2010 ▸) studied 2 × 2 × 2 superstructures of mullite with vacancy concentrations of 0.125, 0.250 and 0.375. Different vacancy distributions were used and the most stable models, *i.e.* the ones with the lowest total free energy, were used for structural investigations. They identified a mechanism for how the presence of vacancies affects the orientation of octahedra. Al/Si ordering was not addressed in the study and seems to be randomly chosen. Aryal *et al.* (2012 ▸) studied different superstructures of mullite with vacancy concentrations 0.25, 0.40, 0.67 and 0.81. The structural models were represented graphically and by pair distribution functions, but from the information provided it is difficult to identify a specific vacancy distribution. It seems that crystal chemical constraints like the avoidance of face-sharing tetrahedra were not considered as the study focused mainly on physical properties. In both studies the satellite reflections and superspace symmetry were not considered to support the generation or analysis of structural models.

**Figure 2:**
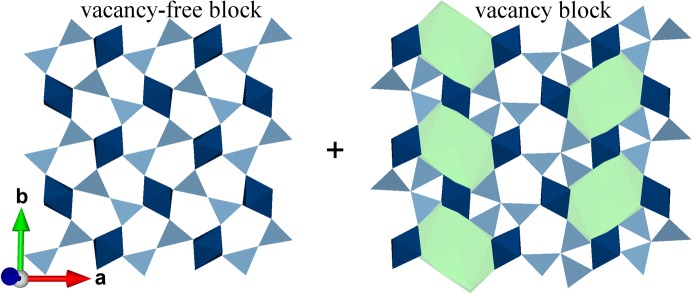
Construction of mullite supercells by combining vacancy-free blocks and vacancy blocks with a composition-adapted length. The shown vacancy block has a length *N*_V_ of two vacancies. Along **c** vacancy blocks are stacked upon vacancy-free blocks and *vice versa*.

**Figure 3:**
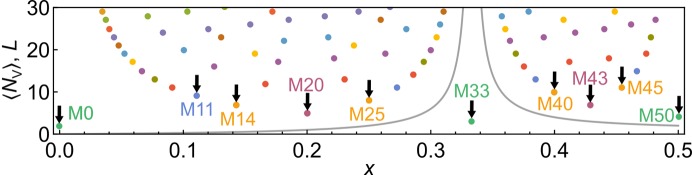
Supercell length *L* (coloured points) and average number of vacancies per vacancy block 〈*N*_V_〉 (solid line) as a function of vacancy concentration *x*. Points with the same colour correspond to a series of rational values of α with the same numerator. For example, the bottom series of green points corresponds to α = 1/2 (M0), 1/3 (M33) and 1/4 (M50). Supercells that were used in this study are marked and labelled.

**Figure 4:**
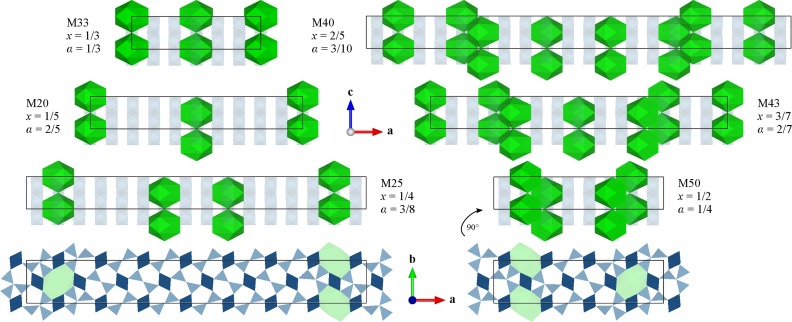
Vacancy distribution patterns of selected mullite supercells. The oxygen coordination spheres of vacancies are shown in green. In views along **b** tetrahedra are neglected for clarity and octahedral chains are shown with decreased opacity. Views parallel to **c** show slabs of the models of M25 (bottom left) and M50 (bottom right). AlO_6_ octahedra are shown in dark blue. Each tetrahedral site is a potential site for Si, but in this figure Al and Si are not distinguished.

Recently, a vacancy ordering pattern in mullite was identified using the superspace approach (Klar *et al.*, 2017*b* ▸). So-called ‘vacancy blocks’ and ‘vacancy-free blocks’ alternate along the **a** and **c** directions (Fig. 2 ▸). The block lengths can be adapted so that superstructures with any vacancy concentration in the range 0 ≤ *x* ≤ 0.5 can be constructed. In the underlying superspace model in the superspace group *Pbam*(α0½)0*ss* the block lengths and the chemical composition are related by the relationship between the modulation wavevector **q** = (α 0 ½) and the vacancy concentration *x*, which is α = (1 − *x*)/2 (Klar *et al.*, 2017*a* ▸). This superspace description allows one to predict the block structure for an arbitrary composition in the range 0 ≤ *x* ≤ 0.5 based on the concept of unified superspace models (Elcoro *et al.*, 2000 ▸; Izaola *et al.*, 2007 ▸). For rational values of *x*, α must also be rational and then the denominator of α determines the length *L* of superstructures with size *L* **a** × 1**b** × 2**c**. The size of each vacancy block can be characterized by the number of vacancies between two vacancy-free blocks counting along the block length. In Fig. 3 ▸ the length *L* and the corresponding mean of the number of vacancies, 〈*N*_V_〉, of commensurate cases are plotted against *x*. If 〈*N*_V_〉 is integer, then 〈*N*_V_〉 = *N*_V_ and all vacancy blocks contain the same number of vacancies. In terms of the vacancy distribution two ranges can be distinguished. For 0 < *x* < 1/3 vacancy blocks are shorter than vacancy-free blocks. For 1/3 < *x* ≤ 0.5 it is the other way around and vacancy blocks are longer than vacancy-free blocks. As a result, mullite superstructures with *x* < 1/3 contain dicluster chains extending along **c** like the dicluster chains in sillimanite. In superstructures with *x* > 1/3 all diclusters either alternate with vacancies or with triclusters. The vacancy concentration *x* = 0.5 marks the endmember because the block pattern cannot be extended to higher vacancy concentrations. In the special case *x* = 1/3 the vacancy blocks and vacancy-free blocks have an infinite length along **a** and **b** which turns the block structure into a layer structure perpendicular to **c** with alternating vacancy layers and vacancy-free layers (Fig. 4 ▸). The description of mullite with *x* = 0 is not identical to sillimanite and therefore sillimanite should not be regarded as an endmember of the mullite solid solution range.

In the range *x* ≤ 0.5 there are 30 cases with *L* ≤ 20. As the number of atoms, *N*_A_, in each supercell amounts to *N*_A_ = 2*L*(16 − *x*) the selected composition range can be studied efficiently with relatively small supercells, which is advantageous for the computational time. Irrational values of *x* correspond to incommensurately modulated structures and were not considered for this study.

## 2. Methodology   

### 2.1. Generation of input structures and force-field calculations   

Structural parameters of 2/1-mullite (*x* = 0.4) and 5/2-mullite (*x* = 0.5) described in space group *Bb*2_1_ *m* are available in the literature (Klar *et al.*, 2017*b* ▸; Kahn-Harari *et al.*, 1991 ▸). As this study has the objective to investigate a broader composition range (*x* ≤ 0.5), a set of ten superstructures was generated (Table 1 ▸). Each supercell with a composition-dependent vacancy distribution is labelled as capital M followed by the first two decimal places of the rounded vacancy concentration, *e.g.* M40 for *x* = 0.4. At first, the vacancy distribution is defined based on the unified superspace model described in Section 1.2 resulting in a block model of vacancy blocks and vacancy-free blocks. In the second step (Fig. 4 ▸), triclusters and diclusters are distributed according to the vacancy distribution with atom coordinates derived from the average atom positions taken from Klar *et al.* (2018 ▸). In the last step, the appropriate amount of Si atoms must be distributed over the tetrahedral sites, whereas all remaining cations are Al. Apart from the analysis of the modulated volumes of tetrahedra, there are only qualitative indications of which sites are favoured by Al and which sites are favoured by Si (Klar *et al.*, 2018 ▸). Therefore, we decided to systematically investigate all symmetry-compliant Al/Si ordering patterns for M0, M25, M33, M40 and M50 with FF methods. In total 46 814 supercells were geometrically optimized (static relaxation) with the GULP code (Gale, 1997 ▸) applying the force model given by Matsui (1996 ▸). These FF parameters proved to give acceptable results for many silicate systems, including sillimanite, andalusite and mullite (Matsui, 1996 ▸; Lacks *et al.*, 2005 ▸; Chen *et al.*, 2008 ▸; Adabifiroozjaei *et al.*, 2018 ▸). The main parameter of interest of the FF calculations was the total energy of a certain Al/Si ordering pattern to evaluate its stability. Several models, mainly the most stable ones, were subsequently used as input structures for DFT calculations.

### 2.2. Parameters of DFT calculations   

DFT calculations were carried out using the ‘*Vienna Ab initio Simulation Package*’ (*VASP*) code (Kresse & Furthmüller, 1996 ▸; Kresse & Joubert, 1999 ▸). Exchange and correlation effects were parameterized within the generalized gradient approximation (GGA) using the Perdew–Burke–Ernzerhof functional (PBE) or the PBEsol variant optimized for solids (Perdew *et al.*, 1996 ▸, 2008 ▸). Calculations for which the dispersion correction after Grimme *et al.* (2010 ▸) was applied are labelled ‘PBE-D’ or ‘PBEsol-D’. In all cases the same set of ultrasoft pseudopotentials provided by the *VASP* program was used. We used a Monkhorst–Pack grid (Monkhorst & Pack, 1976 ▸) for orthorhombic lattices and a gamma-centred *k* mesh for hexagonal lattices (quartz and corundum). Wavefunctions were represented in a plane-wave basis truncated at 520 eV. For each structure several short optimizations were performed at the beginning to redefine the set of plane waves according to the new cell size until the initial pressure was less than 10 kbar (1 kbar = 100 MPa).

Hay *et al.* (2015 ▸) underlined the importance of dispersion correction for DFT calculations on SiO_2_ polymorphs for the correct relative stability because calculations without dispersion correction indicated that cristobalite was more stable than quartz in contrast to experimental observations. Demichelis *et al.* (2010 ▸) reported that the relative stability of the Al_2_SiO_5_ polymorphs is correctly calculated with the PBEsol functional but not with PBE. Initially, our calculations were based on the PBE functional without dispersion correction. Test calculations on mullite have shown that the relative stability of different Al/Si distributions is not affected by the choice of functional. We thus decided to use the PBE functional for the qualitative determination of the most stable Al/Si ordering pattern (Section 3). For these calculations the standard grid for the fast Fourier transform (FFT), a convergence criterion for the forces of 0.02 eV Å^−1^ and a spacing between *k* points of less than 0.017 Å^−1^ was used. More precise calculations with dispersion correction were carried out for the most stable superstructures and for the quantitative comparison with experimental results (Section 4). For those calculations a denser FFT grid was chosen, the convergence criterion for the forces was reduced to 0.01 eV Å^−1^ and the *k*-point spacing was less than 0.014 Å^−1^. For benchmark and comparison purposes the different functionals were used for DFT calculations of the experimentally well-defined structures of α-quartz (α-SiO_2_, Table 2 ▸), corundum (α-Al_2_O_3_, Table 3 ▸) and the three Al_2_SiO_5_ polymorphs sillimanite, andalusite and kyanite (Tables S1, S2 and S3 in the supporting information).

## 3. Determination of superspace models from first principles   

### 3.1. Ideal Al/Si ordering pattern   

**Figure 5:**
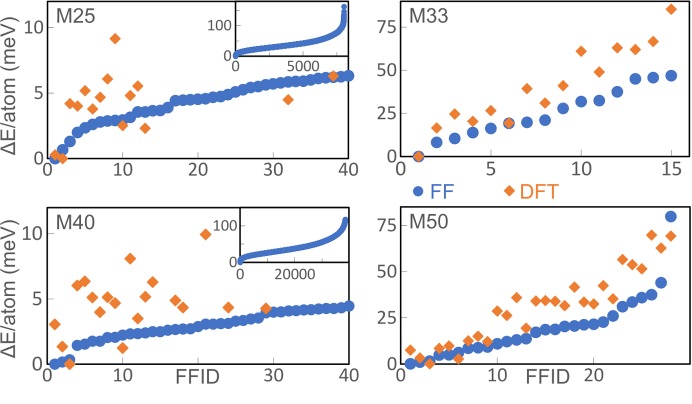
Relative total energies of geometrically optimized supercells with respect to the most stable supercell. The horizontal axis (FFID) is the permutation number which is identical to the rank determined by FF calculations (blue circles). The relative total energies per atom determined by DFT calculations (orange diamonds) are expressed relative to the total energy of the supercell which according to the DFT calculations is most stable (#1).

As the ideal distribution of Si atoms in mullite was not known, we assumed that the Al/Si ordering adopts the highest possible symmetry, *i.e.* the orthorhombic symmetry of the block structures (Fig. 4 ▸) is not reduced by the Al/Si ordering. The ideal superstructure then corresponds to the structure with the lowest energy. For M0, M33 and M50 the supercells are relatively small and therefore there are only a few Al/Si permutations. For these cases all Si distributions were investigated with FF and DFT calculations. Each supercell was assigned a number (FFID) corresponding to its stability according to the FF calculations. A comparison with an analogue ranking based on the DFT calculations allows one to evaluate the accuracy of the FF calculations. The ranking of M0 is identical with both approaches, *i.e.* in this case the FF calculations correctly identify the most stable and least stable Al/Si ordering patterns. The FFID ranking of M33 deviates by about one position relative to the DFT ranking (Fig. 5 ▸). However, the first and last FFID correctly represent the most and least stable supercell, respectively. In the case of M50 the uncertainty of the ranking is about two positions and neither the first nor the last rank are correctly identified. The geometric optimization of the least stable structures with DFT in many cases results in a strong deformation leading to larger deviations from the bond lengths expected from X-ray diffraction studies. In contrast, the coordinates of the most stable structures are only slightly displaced from the expected coordinates. These results are taken as an indication that a qualitative assessment of the relative stability of a certain supercell is possible with the FF calculations, but for the determination of the ideal Al/Si ordering more accurate DFT calculations must be carried out.

For M25 and M40 there are 8008 and 38 760 Al/Si permutations, respectively, and the computational cost to study all of them with DFT is very high. Therefore, all supercells were geometrically optimized with FF methods and assigned an FFID according to the respective total energy. The most stable supercells (low FFIDs) were considered for DFT calculations, although additional calculations were carried out for a better overview. Fig. 5 ▸ compares graphically the relative energies of M25, M33, M40 and M50. Absolute total energies, relative energies and selected characteristics of the Al/Si ordering pattern are given in the supporting information in Table S4. To identify different supercells the rank as determined by the DFT calculations is given with the hashtag symbol. For example, the most stable supercell of M50 considered to show ideal Si distribution is labelled M50 #1.

A qualitative comparison of the different Al/Si ordering schemes allows one to establish empirical rules to estimate the stability of a certain Si distribution. M0 #3, the least stable supercell, contains Al_2_O_7_ units (Al–Al diclusters), in which the bridging oxygen is strongly underbonded (SUBO) according to Pauling’s bond valence rules (Pauling, 1929 ▸; Loewenstein & Lowenstein, 1954 ▸). M0 #2 contains strongly overbonded oxygen (SOBO) atoms which are shared by two SiO_4_ tetrahedra and one AlO_6_ octahedron. These two structural elements are also present in the least stable supercells of M33 and M50. None of the M25 and M40 supercells investigated with DFT contains SOBOs and only one supercell of M25 contains Al–Al diclusters with SUBOs (FFID 9). In M25, M33 and M40 there are dicluster–vacancy columns, *i.e.* diclusters alternate with vacancies parallel to **c**, and in the ideal permutations these diclusters are consistently of the Si–Si type. In general, it is assumed that Si does not occupy tetrahedral sites of triclusters due to the anion charge on the threefold coordinated oxygen (Pauling, 1929 ▸). Si is expected to especially avoid the T* site of triclusters as the experimentally observed bond lengths are too long for typical Si—O distances (Angel *et al.*, 1991 ▸; Schneider *et al.*, 2015 ▸). M40 #1 and M50 #1 contain Si in triclusters indicating that this is a stable and favourable environment for Si. The identification of Si in triclusters and the presence of Si–Si diclusters between vacancies are consistent with the Al/Si ordering pattern suggested by Klar *et al.* (2018 ▸). As M40 #2 and M50 #2 contain Si on the T* site, the presence of this tricluster configuration is energetically not forbidden (Table S4).

The mentioned structural elements only take the direct environment of selected sites into account and less localized interactions, *e.g.* the relative orientation of neighbouring Al–Si diclusters or its effect on the distortion of octahedra, are not analysed here. The analysis of the local environment should only be considered as a rough estimate of the stability of a certain Al/Si ordering pattern because these criteria are not enough to characterize the crystal chemistry of a certain Al/Si permutation. For example, M50 #18 is rather unstable but does not contain Al–Al diclusters or strongly overbonded oxygens.

On the other hand, a full parameterization of the Al/Si ordering of a certain supercell is rather cumbersome to set up, analyse and describe. This becomes even more complicated if different supercells with a different number of atoms are compared. Hence, the comparison of total energy calculations of supercells described in physical space is a useful method to systematically determine the ideal Al/Si ordering pattern, but a comparison and assessment of the resulting supercells are inconclusive. An elegant and efficient solution to this problem is to compare the structures in (3+1)-dimensional superspace, which requires the establishment of superspace models from the geometrically optimized structures.

### 3.2. Superspace models based on DFT calculations   

The geometrically optimized models can be used to determine displacive and occupational modulation functions. It is a common step to represent physical space sections of (3+1)-dimensional superspace models, especially for visualization purposes. These sections are subspaces of superspace perpendicular to the fourth axis **a**_s4_. Different axis intercepts correspond to different physical space models, which in incommensurate cases only differ by a structurally irrelevant origin shift. Structural aspects of all sections are analysed in *t* space, where *t* is defined by the axis intercept. To determine the modulation function from the supercell coordinates the atoms of the supercell are embedded in a (3+1)-dimensional unit cell defined by the modulation wavevector **q** and a basic unit cell (Pérez-Mato *et al.*, 1991 ▸; Izaola *et al.*, 2007 ▸; Orlov *et al.*, 2008 ▸).

**Figure 6:**
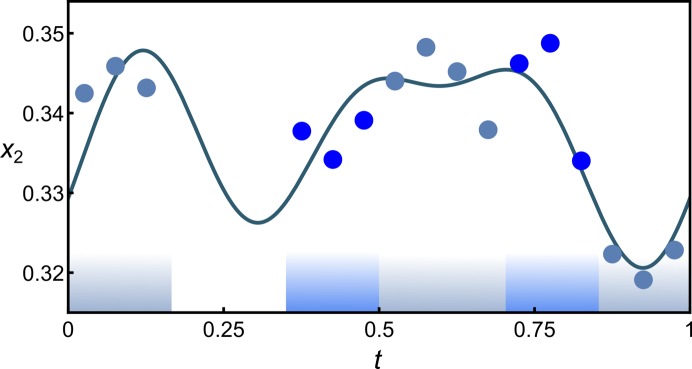
*x*_2_ of *T* site of M40 #1 (PBE). Grey-blue corresponds to Al, dark blue to Si. Fitted modulation functions with first- to third-order harmonics are shown as a solid line. Approximate *t* sections at which the block wavefunctions of the occupational modulation of Al2 and Si2 are valued 1 are indicated at the bottom.

In mullite the *T* site is of major interest as Al/Si ordering mainly takes place on that site and it participates in both diclusters and triclusters. In the following example, the modulation functions describing the atomic domain of the *T* site are determined for M40 #1. Fig. 6 ▸ shows the *x*_2_ coordinates of Al2 (grey-blue) and Si2 (blue) atoms in *t* space and the determined modulation function 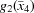 defined as
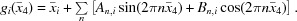


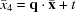
 is the dot product of the modulation wavevector **q** and the average coordinate vector 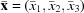 plus the parameter *t* defined by the physical space section. *n* is the order of the harmonic term. The parameters that must be determined by least-square fitting to the embedded coordinates are the average coordinate 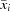 and the component amplitudes *A*_*n*,*i*_ and *B*_*n*,*i*_. Symmetry restrictions decrease the number of parameters for atoms occupying special positions. For example, a modulation of *x*_3_ is forbidden for all atomic domains except Al1 and O2. The displacive modulation functions can be determined for any atomic domain if sufficient coordinates of a specific average structure site are available. Here, there are four independent coordinate triplets for Al3 and O4 and 20 for Al1, O1 and O2. In the present example, meaningful modulation functions can be determined if the observables-to-parameter ratio for the fitting is larger than about 1.5. In Fig. 6 ▸ the determined modulation function of *x*_2_ of the *T* site is shown with harmonics of order 1 to 3.

**Figure 7:**
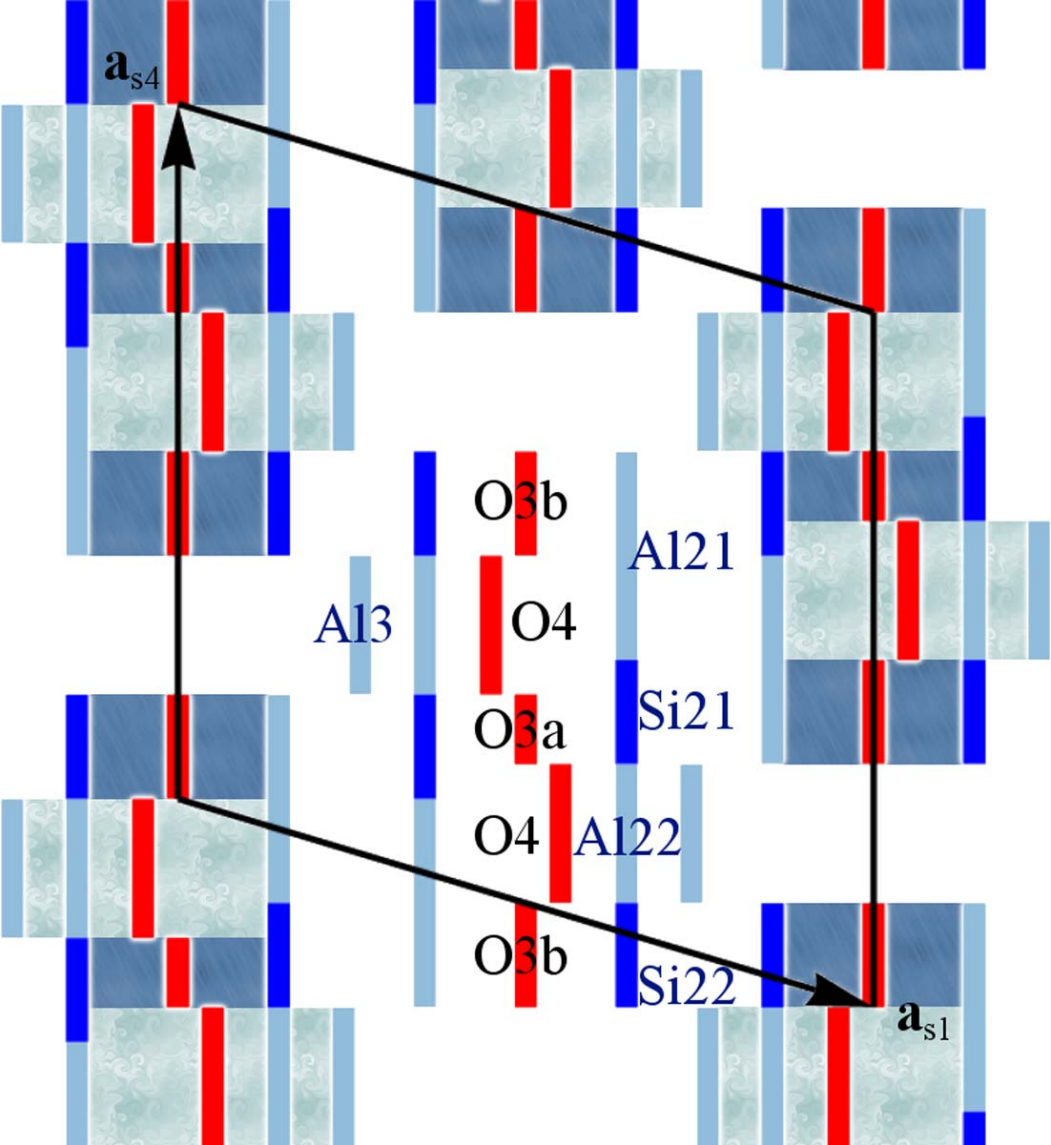
Section of the superspace model based on M40 #1 showing atomic domains relevant for Al/Si ordering. Displacive modulations are neglected here. Atomic domains that form part of the octahedra, *i.e.* Al1, O1 and O2, are not shown. Different background colours indicate dicluster and tricluster units. The domains of Al2, Si2 and O3 are described by more than one block wavefunction.

Because of the nature of the computational methods the occupancy of atoms is either 0 or 1. Respective occupational modulation functions are described as block wavefunctions defined by a block wave centre 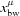 and a length 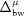 (Petríček *et al.*, 2016 ▸):
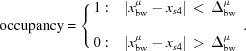



The occupational modulation functions of O3 and O4, which define the locations of diclusters and triclusters, were already known from the initial model [Table S1 in Klar *et al.* (2017*b* ▸)]. The characterization of the Al/Si ordering in superspace thus requires the determination of 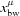 and 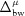 of Al and Si on the *T* site, which is the missing information provided by this work. Fig. 6 ▸ suggests that four block wavefunctions are required to describe the occupational modulation of Al2 and Si2. The block wave parameters of the atomic domains of Al21, Al22, Si21 and Si22 (Fig. 7 ▸) can only be determined with a precision that is intrinsically limited by the number of independent coordinates of the commensurate case. However, in combination with the known occupational modulation parameters of O3 and O4 (Klar *et al.*, 2017*b* ▸) the exact parameters to describe the specific Al/Si ordering scheme can be determined because in the present example Si22 is always bonded to O3b (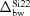 = 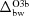, 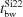 = 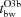) and Al22 only occurs in triclusters together with Al3 (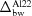 = 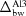, 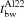 = 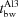). 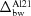 is twice 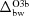 and 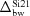 = 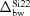. Thus, all block wave parameters are exactly determined (Table 4 ▸). The embedding of M40 #1 and the characterization of the modulation functions (Fig. 6 ▸) thus allowed us to define the Al/Si ordering pattern in superspace as shown in Fig. 7 ▸.

Together with the displacive modulation functions all but thermal displacement parameters for the description of a superspace model are known. Methods to determine ADPs from phonon calculations are available, but were not applied here (Madsen *et al.*, 2013 ▸).

### 3.3. Unified superspace model   

A unified superspace model without Al/Si ordering was suggested by Klar *et al.* (2017*a* ▸) for the range 0 ≤ *x* ≤ 0.5. Combining this suggested model with the approach of Section 3.2 and the comparison of this section allows us to define the occupational modulation parameters as a function of the vacancy concentration *x*. Thus, a unified superspace model is established assuming that there is a unique ideal Al/Si ordering pattern describing mullite with 0 ≤ *x* ≤ 0.5.

**Figure 8:**
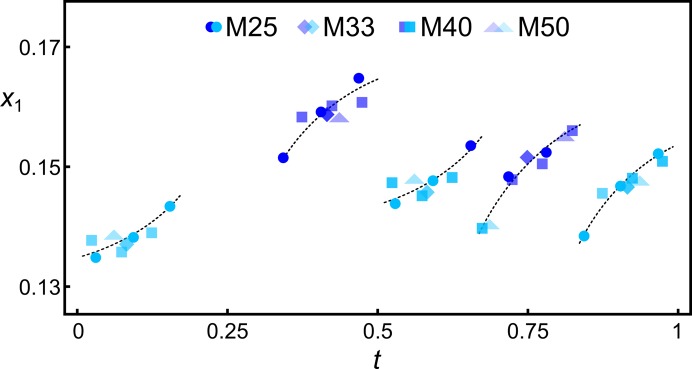
Coordinate *x*_1_ of tetrahedral Al2 (light blue) and Si2 (dark blue) atoms of geometrically optimized (PBE) superstructures with labels M25 #2 (circles), M33 #1 (diamonds), M40 #1 (squares) and M50 #1 (triangles). The displacive and occupational modulation functions follow the same trend, allowing a description of the solid solution range by a unified superspace model. Dashed lines indicate trend lines fitted by hand.

In Section 3.1 it was mentioned that a characterization of different Al/Si ordering patterns for comparison in physical space is not straightforward to assess. Modulation functions, bond lengths and other parameters of crystal chemical interest can be easily compared in superspace independent of the composition. A comparison of the Al/Si ordering patterns of M25 #2, M33 #1, M40 #1 and M50 #1 shows that the modulation functions of Al2 and Si2 on the *T* site are very similar in terms of the displacive modulation, the number of required atomic domains and the occupancy block wave parameters (Fig. 8 ▸). This simple comparison of atomic domains directly indicates that the Al/Si ordering pattern for the different compositions is the same in superspace and thus the rules for how Al and Si are ordered in physical space are also the same. Here, the focus lies on the occupational modulation and the displacive modulation is not further considered for the sake of simplicity. The parameters of the block wavefunction as a function of the composition are defined in Table 4 ▸. The use of M25 #2 instead of M25 #1 is justified by the small energy difference of 0.3 meV/atom between the two ordering schemes. Furthermore, M25 #1 requires three Al and three Si domains to describe the *T* site in superspace and the Al/Si ordering pattern cannot be extended to compositions with *x* ≥ 1/3 because it collapses if dicluster chains are not present. An animated figure of the unified superspace model like Fig. 7 ▸ is included in the supporting information.

## 4. Comparison with experimental observations   

In this section these results are compared with experiments. For that purpose, the DFT calculations of the energetically most stable superstructures of Section 3 were repeated with higher precision and applying dispersion correction (PBEsol-D), which is expected to improve the agreement between the calculated and experimental lattice parameters as pointed out in Section 2.2. Furthermore, the unified superspace model of the last section was used to predict additional superstructures with different compositions (M11, M14, M20, M43, M45), including compositions that are expected to be unstable according to the phase diagram (M11, M14). Selected characteristics of the superstructures are given in Table 1 ▸. These calculations with the ideal Al/Si ordering are labelled AS1.

**Figure 9:**
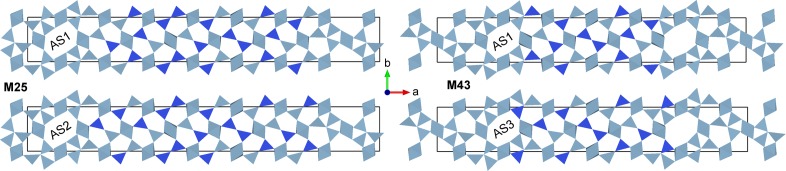
Geometrically optimized (PBEsol-D) structure models of M25 (left) and M43 (right) with different Al/Si ordering patterns (AS1, AS2, AS3).

The Al/Si ordering in superspace of M25 #1 was also determined and implemented for more accurate calculations in the range 0 < *x* < 1/3, which are labelled AS2. This Al/Si ordering scheme is not applicable for *x* > 1/3 as described in Section 3.2. However, the qualitative difference between M25 #1 (AS2) and M25 #2 (AS1) is the orientation of Al–Si diclusters at the borders between vacancy blocks and vacancy-free blocks. This orientation scheme can be implemented in supercells with *x* > 1/3 by adapting the orientation of respective diclusters and triclusters (AS3). Geometrically optimized structural models of M25 (AS1, AS2) and M43 (AS1, AS3) are shown in Fig. 9 ▸ and of M50 (AS3) in the supporting information.

### 4.1. Lattice parameters   

**Figure 10:**
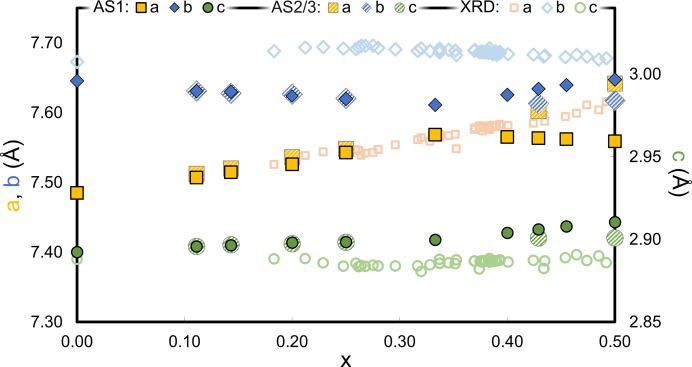
Lattice parameters of geometrically optimized supercells (PBEsol-D) for Al/Si ordering patterns AS1, AS2 (*x* < 1/3) and AS3 (*x* > 1/3). Experimental values of *a*, *b* and *c* based on X-ray diffraction measurements are taken from Cameron (1977*a* ▸), Klug *et al.* (1987 ▸), Ban & Okada (1992 ▸) for mullite and from Yang *et al.* (1997 ▸) for sillimanite (*x* = 0). Several experimental values of *a* are ‘hidden’ behind the data points of the DFT calculations.

Lattice parameters of all geometrically optimized supercells were extracted. The parameter *a* was divided by *L* and *c* was divided by 2 for comparison. The resulting parameters of all compositions and the different Al/Si ordering patterns are shown in Fig. 10 ▸. Experimental parameters were included as described in the figure caption. Experimental lattice parameters were measured at ambient conditions, whereas the calculations correspond to a temperature of 0 K and a pressure of 0 Pa. No measurements of thermal expansion coefficients or lattice parameters close to these conditions are available, but the extrapolation of measurements at elevated temperatures (Schneider & Eberhard, 1990 ▸) allows one to estimate that a temperature correction would decrease the experimental *a* and *c* by less than 0.01 Å and *b* by less than 0.02 Å. This has no relevant influence on the present analysis.

The lattice parameters of AS1 depend linearly on the vacancy concentration *x*, but two realms with different slopes are observed. For *x* < 1/3 the parameter *a* increases linearly with *x*, resembling experimental observation remarkably well. In the same realm *b* decreases slightly, but neither the slope nor the absolute values agree with experimental observations. For higher vacancy concentrations (*x* > 1/3), *a* slightly decreases and deviates from the experimental observations. *b* increases but still shows a clear offset from the reference values. The lattice parameters are almost unaffected by changing the orientation of the Al–Si dicluster chains because the parameters with the AS2 pattern (*x* < 1/3) are almost identical with those of AS1. *c* varies very little over the whole composition range and is hardly affected by the different Al/Si ordering patterns. Despite the good overlap of *a* for *x* < 1/3, the overall picture indicates that the model does not account for all relevant structural aspects. This is not surprising because the calculations are based on fully ordered models with strict lattice periodicity along **b** whereas the real structures are disordered. Investigations of the diffuse scattering revealed that important inter-vacancy vectors are, among others, [1.5 0.5 0] and [0.5 1.5 0] (Freimann & Rahman, 2001 ▸). The first also plays a crucial role in all supercells with 〈*N*_V_〉 > 1, but [0.5 1.5 0] breaks the lattice periodicity along **b**. Therefore, it is likely that the ordered model is an appropriate approximation of the disordered vacancy distribution along **a**, but not along **b** which explains the observed offset between *b* from DFT calculations and experiments. It is unlikely that disorder in the real structure is limited to vacancies as Al/Si disordering can be expected as well. The lattice parameters of AS3 show that small changes of the Si distribution (Fig. 10 ▸) result in a significant change of the lattice parameters. The discrepancy in the determined values of *a* is thus likely to originate from Al/Si disorder. Therefore, the unified superspace model is considered to describe the general ordering pattern of vacancies and Al/Si, but in the real structure these patterns are not strictly followed. This is supported by the low energy differences between similar Al/Si ordering patterns (Section 3.1). More work is needed to better understand the disorder in the real structure for different degrees of ordering (Klar *et al.*, 2018 ▸).

### 4.2. Stability of the mullite phase   

**Figure 11:**
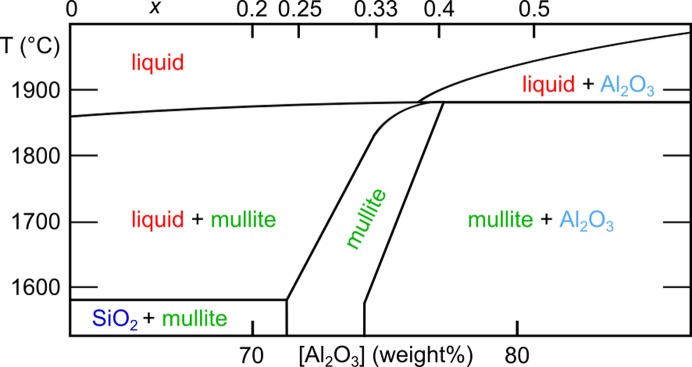
Part of the SiO_2_–Al_2_O_3_ phase diagram adapted with permission from Klug *et al.* (1987 ▸). Copyright (1987) The American Ceramic Society.

According to the SiO_2_–Al_2_O_3_ phase diagram presented by Klug *et al.* (1987 ▸) the width of the solid solution range corresponds to a vacancy concentration difference of about 0.06. The borders of the range shift to higher vacancy concentrations with increasing temperature. Close to the melting point the width of the solid solution range becomes very narrow around *x* ≃ 0.4. A part of the phase diagram is sketched in Fig. 11 ▸, in which the composition is also indicated by the vacancy concentration. The temperature dependence agrees with other studies on mullite formation from mineral decomposition (Rinne, 1924 ▸; Schneider & Majdič, 1980 ▸; Holm, 2001 ▸; Rahman *et al.*, 2001 ▸) and especially mullite–mullite transformations at higher temperatures (Cameron, 1976 ▸; Schmücker *et al.*, 2002 ▸), although other phase diagrams were suggested with a negligible dependence of the mullite composition on the temperature of formation (Aksay & Pask, 1974 ▸).

The unified superspace model explains many details of the structural dependence on the composition and presents the underlying mechanisms of vacancy and Al/Si ordering. Some of the commensurate cases investigated here represent structural turnover points: for *x* < 0.2 the block structure begins to dissolve as a certain fraction of vacancy blocks disappears. *x* = 1/3 is a special case because of its layer structure and *x* = 0.5 marks the endmember of the unified superspace model for which dicluster–vacancy chains disappear. For *x* > 0.5 a new superspace model is required, in full agreement with the observation of a lowering of the symmetry to monoclinic above *x* ≃ 0.5 (Ylä-Jääski & Nissen, 1983 ▸). Independent of the superspace model tetraclusters are present for *x* > 2/3. The observed borders of the solid solution range do not correspond to any of the above-mentioned turnover compositions. Hence, the vacancy distribution predicted by the superspace model cannot explain the phase diagram and a different explanation is needed.

**Figure 12:**
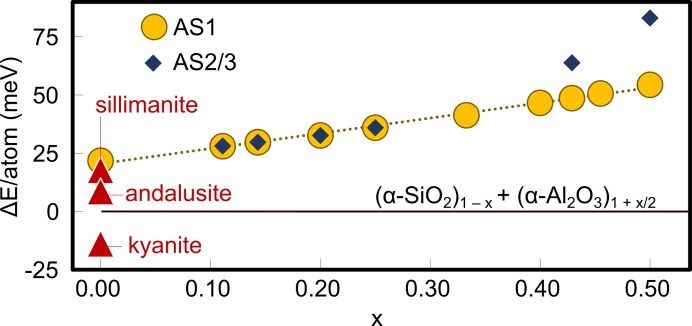
Comparison of calculated total energies (PBEsol-D) of aluminium silicates with total energies of a hypothetical system where the atoms form respective amounts of α-SiO_2_ and α-Al_2_O_3_.

In the following the total energies of the DFT calculations are used to further investigate the solid solution range of mullite. It is not straightforward to compare the energies of different systems if the chemical composition is not identical. The energy contributions of the vacancy and Al/Si distribution of mullite were isolated from the chemical composition by subtracting the total energy of a supercell from a chemically identical system consisting of α-SiO_2_ and α-Al_2_O_3_. In Fig. 12 ▸ this energy difference is plotted against the vacancy concentration. The main observation is that the relative stability of mullite decreases with increasing vacancy concentration. The energies of AS1 and AS2 are essentially identical, whereas AS3 is significantly less stable than AS1. However, according to the calculations only kyanite is more stable than a chemically identical mixture of α-SiO_2_ and α-Al_2_O_3_ and any mullite supercell is less stable. Interestingly, the linear dependence of the energy on the vacancy concentration seems to be independent of the block lengths, indicating that the vacancy concentration or chemical composition is more important than the actual vacancy distribution. This may explain why in mullite the tendency for long-range vacancy ordering is not very pronounced.

According to the energy analysis mullite tends to avoid the formation of vacancies, and the linear dependence on the composition also confirms that none of the turnover compositions is energetically favoured in agreement with the above conclusions. Hence, the observed stability of mullite must be related to the kinetics and structural dynamics at the temperature of formation. Although all calculations of this study neglect any dynamics arising from temperature or pressure, some indications on the dynamics can be derived from structural elements of the unified superspace model: it may be expected that Si tetrahedra are the most rigid structural units in mullite. This is supported by the volume of the Si tetrahedra forming part of the Si–Si diclusters in the relaxed structures. The smallest volume and largest volume are 2.296 Å^3^ (M11 AS1) and 2.304 Å^3^ (M45 AS1), respectively. In Section 3.1 it was shown that Si–Si diclusters are preferably found stacked between vacancies, indicating that the geometry of vacancies may adapt effectively to the requirements of other structural units. This effect is likely to be more pronounced at higher temperatures. Then, the stability of mullite will depend mainly on the vacancy concentration and the temperature, but less on the actual vacancy distribution, in agreement with the above-mentioned observations. In this picture, the silica-rich border of the solid solution range is defined by the low-temperature limit at which mullite forms under equilibrium conditions. Even lower vacancy concentrations cannot form because the corresponding vacancy concentrations require a temperature at which the formation of mullite is not observed, which explains the miscibility ‘gap’ for *x* < 0.2. The alumina-rich border in turn corresponds to the vacancy concentration that is most stable at temperatures just below the melting point. Higher vacancy concentrations are achievable by quenching melts from *e.g.* 2100°C if the presence of α-Al_2_O_3_ nuclei can be avoided (Cameron, 1977*b* ▸; Kriven & Pask, 1983 ▸; Aksay *et al.*, 1991 ▸). Increasing the pressure decreases the structural flexibility of mullite and above 2 kbar it decomposes into Al_2_SiO_5_ and α-Al_2_O_3_ (Schneider & Komarneni, 2005 ▸) in agreement with our interpretation of the present results. Although the suggested mechanism is consistent with many experimental observations, a deeper structural and thermodynamic study should be carried out for verification and also to investigate the role of entropy. We believe that the results offer a good starting point for a study applying molecular dynamics to finally understand better the stability and high-temperature applications of mullite (Aksay *et al.*, 1991 ▸; Schneider *et al.*, 2015 ▸).

### 4.3. Comparison of computed models with refinements   

Several electron diffraction studies have reported on mullite with high-order satellite reflections (Nakajima & Ribbe, 1981 ▸; Ylä-Jääski & Nissen, 1983 ▸; Wang *et al.*, 2007 ▸). Structural models were not refined, but Klar *et al.* (2018 ▸) have shown that different degrees of order exist and that with the same constraint scheme the occupational modulation functions of partially and highly ordered mullite can be described. To date, only refinements of disordered structures based on first-order satellite reflections using modulation functions with first-order harmonics have been published (Birkenstock *et al.*, 2015 ▸; Klar *et al.*, 2017*b* ▸, 2018 ▸). A superspace model refined against synchrotron single-crystal X-ray diffraction measurements (SA1 in Klar *et al.*, 2018 ▸) was chosen as experimental reference for comparison with the superspace model of M40 AS1 (PBEsol-D) determined with the method presented in Section 3.2. At first glance, the modulation functions from the refinement show a significantly weaker amplitude. This is to be expected as the calculations are based on a maximally ordered model and the refinement is based on a disordered structure with weak low-order satellite reflections. Therefore, the following modifications were applied: from the fitted modulation functions containing up to third-order harmonic terms only first-order harmonic terms were used and higher harmonics were cut off. The refinement modulation functions were amplified by a scale factor *s* determined by minimizing the residual *R* factor defined as
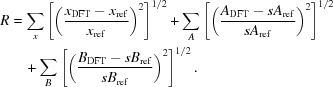

**Figure 13:**
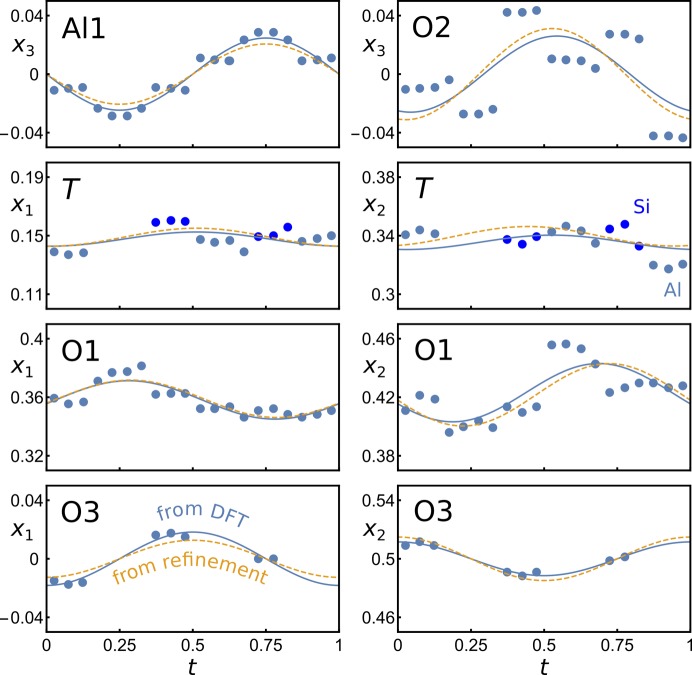
Figure comparing amplified modulation functions from refinement [dashed line, Klar *et al.* (2018 ▸)] with harmonic fits (solid lines) of M40 #1.

*x*, *A* and *B* are the variables in 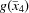 determined by the least-squares fitting for *x*_1_ and *x*_2_ of *T*, O1 and O3, *x*_3_ of Al1 and O2 and the respective *A* and *B* amplitudes with *n* = 1. Because of symmetry constraints only four coordinates, eight *A* and five *B* values must be determined. The atomic domains of Al3 and O4 were not considered in this procedure due to the low observables-to-parameters ratio and the fact that the four coordinate triplets are concentrated in a limited range in *t* space. The minimum *R* = 0.02 is found if *s* = 4.4. In Fig. 13 ▸ the amplified modulation functions are shown together with the embedded coordinates and the calculated modulation functions from the fitting procedure.

The agreement between the calculated modulation functions and amplified refined modulation functions is excellent for the displacive modulation of *x*_1_ of the *T* site and O1, but in turn the agreement is worse for the respective modulations of *x*_2_. The overall residual *R* factor = 0.02 indicates that the calculations are in good agreement with experimental observations and that the method applied here allows one to establish and validate superspace models for comparison with or as reference for experimental observations.

## 5. Summary and conclusions   

The benefit of first-principles calculations for the investigation of modulated structures was demonstrated using the example of mullite. A systematic study based on FF and DFT calculations on supercells of different compositions and with different Si distributions allowed us to determine for the first time the details of the ideal Al/Si ordering pattern in mullite for vacancy concentrations 0 ≤ *x* ≤ 0.5. Quantitative modulation functions describing the displacive and occupational modulation were determined and compared, which indicated that the ordering mechanisms for all compositions are the same. On this basis a unified superspace model for the investigated solid solution range of mullite was established. A comparison with modulation functions based on X-ray diffraction experiments indicates that the applied method correctly determined the underlying Al/Si ordering pattern of the most ordered state of mullite. The sole energetical analysis of the solid solution range cannot explain the phase diagram of mullite. Nevertheless, a mechanism focusing on the structural flexibility due to the presence of oxygen vacancies was suggested to explain the observed stability range of mullite. Klar *et al.* (2018 ▸) showed that the superspace group *Pbam*(α0½)0*ss* allows one to derive the vacancy distribution pattern in mullite. As a consequence, the average structure and the superspace symmetry are sufficient to establish a superspace model from first principles.

This example shows that many different aspects of a complex, modulated structure family can be studied based on the determination of superspace models from first principles as introduced in this study. The method is generally applicable to any modulated structure for which a representative commensurate case or commensurate approximation can be generated. In many cases X-ray diffraction does not allow the unambiguous interpretation of features with weak contrast like the position of atoms with very few electrons or the distinction between atoms with similar scattering power like Al and Si (Xu *et al.*, 2016 ▸). These questions can be tackled by following the systematic approach based on FF calculations and more accurately with DFT calculations. Although the present example was based on commensurate cases, we do not see any obstacle to ‘extrapolating’ the modulation functions for the description of incommensurately modulated structures.

As more powerful algorithms and computers are developing, the number of atoms in the system becomes less relevant for the consideration of DFT calculations. In particular, as linear-scaling DFT codes are well established, calculations with thousands of atoms are not an obstacle any more (Goedecker, 1999 ▸; Soler *et al.*, 2002 ▸; Mohr *et al.*, 2018 ▸). Hence, input structures for calculations on modulated structures should not be as simple as possible, but rather as complex as necessary to allow the investigation of the modulation from first principles.

## 6. Related literature   

For additional literature relating to the supporting information, see Burt *et al.* (2006 ▸) and Winter & Ghose (1979 ▸).

## Supplementary Material

Additional tables and figures. DOI: 10.1107/S2053273319000846/gv5003sup1.pdf

Animation of the unified superspace model of Section 3.4. See Fig. 7 for labels.. DOI: 10.1107/S2053273319000846/gv5003sup2.gif

## Figures and Tables

**Table 1:** Description of mullite supercells The number of Al/Si permutations is calculated considering only space-group-compliant Al/Si distributions. FF supercells and DFT supercells refer to the number of different, geometrically optimized Al/Si permutations.

Label	Al_2_O_3_/SiO_2_	*x*	α	Supercell	Space group	〈*N*_V_〉	*N*_A_	Al/Si permutations	FF supercells	DFT supercells
M0	1/1	0	1/2	2 × 1 × 2	*Bb*2_1_ *m*	0	64	3	3	3
M11	19/16	1/9	4/9	9 × 1 × 2	*Pnnm*	1/3	286	43758	0	2
M14	5/4	1/7	3/7	7 × 1 × 2	*Pbam*	1/2	222	3003	0	2
M20	11/8	1/5	2/5	5 × 1 × 2	*Pnnm*	1	158	210	0	2
M25	3/2	1/4	3/8	8 × 1 × 2	*Bb*2_1_ *m*	2	252	8008	8008	15
M33	7/4	1/3	1/3	3 × 1 × 2	*Pbam*	∞	94	15	15	15
M40	2/1	2/5	3/10	10 × 1 × 2	*Bb*2_1_ *m*	4	312	38760	38760	21
M43	17/8	3/7	2/7	7 × 1 × 2	*Pnnm*	3	218	1001	0	2
M45	9/4	5/11	3/11	11 × 1 × 2	*Pbam*	5/2	342	74613	0	1
M50	5/2	1/2	1/4	4 × 1 × 2	*Bb*2_1_ *m*	2	124	28	28	28

**Table 2:** Optimized structure parameters of α-SiO_2_ Average bond lengths *d* given in Å. *E* is the total free energy. Reference labels: (N) Cline (2017 ▸), (O) O’Connor & Raven (1988 ▸), (H) Hay *et al.* (2015 ▸).

Reference/functional	*a* (Å)	*c* (Å)	*V* (Å^3^)	*d*(Si^IV^—O)	*E* (eV)
Calculations of this study:					
PBE	5.0203	5.5084	120.23	1.6274	−71.14
PBE-D	4.9509	5.4581	115.86	1.6264	−71.99
PBEsol	4.9489	5.4441	115.47	1.6229	−74.01
PBEsol-D	4.8956	5.4027	112.14	1.6222	−74.69
Experimental powder XRD reference:					
(N) NIST 1878b	4.91378 (30)	5.40536 (30)	113.03		
(O)	4.9070 (6)	5.3997 (4)	112.60	1.5999	
Calculation references:					
(H) PBE	5.031	5.514	120.84	1.616	
(H) TS	4.928	5.428	114.15	1.616	

**Table 3:** Optimized structure parameters of α-Al_2_O_3_ Average bond lengths *d* given in Å. *E* is the total free energy. Reference labels: (N) Cline (2014 ▸), (O) O’Connor & Raven (1988 ▸), (T) Tohei *et al.* (2016 ▸).

Reference/functional	*a* (Å)	*c* (Å)	*V* (Å^3^)	*d*(Al^VI^—O)	*E* (eV)
Calculations of this study:					
PBE	4.8100	13.1231	262.94	1.9330	−224.45
PBE-D	4.7874	13.0612	259.25	1.9240	−229.09
PBEsol	4.7757	13.0158	257.09	1.9182	−236.00
PBEsol-D	4.7600	12.9656	254.41	1.9116	−239.74
Experimental powder XRD reference:					
(N) NIST 676a	4.759355 (80)	12.99231 (15)	254.87		
(O)	4.7585 (6)	12.9824 (6)	254.58	1.9133	
Calculation references:					
(T) PBE	4.807	13.115	262.45		

**Table 4:** Occupational modulation function parameters of the unified superspace model 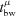
 is the centre of the block wavefunction in *t* space.

Site label	O3a	O3b	O4	*T*	T*	Al21	Al22	Si21	Si22
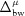	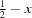	(1 − *x*)/4	*x*/2	1 − *x*/2	*x*/2	(1 − *x*)/2	*x*/2	(1 − *x*)/4	(1 − *x*)/4
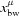	0	(1 + α)/4	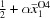	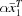	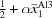	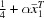	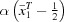	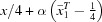	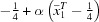
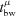	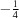	(1 − *x*)/8	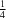	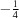	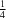	0	(2 − *x*)/4	3(*x* − 1)/8	(*x* − 5)/8
