# Supplementary material for: Characterizing modulated structures with first-principles calculations: a unified superspace scheme of ordering in mullite
Source: Acta Crystallogr A Found Adv. 2019 Feb 12;75(Pt 2):260–72. doi: 10.1107/S2053273319000846 (PMC6396396; doi:10.1107/S2053273319000846)
Supplement: Supplementary file 1 [file a-75-00260-sup1.pdf]

Acta Cryst A FOUNDATIONS ADVANCES

Volume 75 (2019)

Supporting information for article:

Characterizing modulated structures with first-principles calculations: a unified superspace scheme of ordering in mullite

Paul Benjamin Klar, Iñigo Etxebarria and Gotzon Madariaga

S1. Additional results of DFT calculations

Table S1 Optimized structure parameters of sillimanite

Average bond lengths *d* given in Å. *E* is the total free energy. Similar shading colours indicate that similar results are expected as similar methods were used.

Reference / Functional	*a* (Å)	*b* (Å)	*c* (Å)	*V* (Å^3)	*d*(Al^{VI}-O)	*d*(Al^{IV}-O)	*d*(Si^{IV}-O)	*E* (eV)
Calculations of this study:								
PBE	7.5718	7.7762	5.8167	342.49	1.933	1.777	1.643	-244.624
PBE-D	7.5428	7.7329	5.8003	338.32	1.924	1.771	1.639	-248.686
PBEsol	7.5164	7.6949	5.7894	334.85	1.915	1.768	1.636	-255.602
PBEsol-D	7.4917	7.6597	5.7755	331.42	1.908	1.764	1.6331	-258.846
Experimental single crystal XRD references:								
(B) 0.0001 GPa	7.48388 (17)	7.6726 (3)	5.76807 (13)	331.21 (2)	1.91	1.76	1.62	
(W) 25 °C	7.4883 (7)	7.6808 (7)	5.7774 (5)	332.29 (5)	1.912	1.763	1.627	
Calculation references:								
(A) PBE	7.4474	7.6014	5.7469	325.34				
(C) PBE	7.557	7.768	5.811	341.11				–246.026
(D) PBE	7.5678	7. 7694	5.8453	343.69	1.934	1.782	1.651	
(D) PBEsol	7.5061	7.6823	5.8159	335.37	1.916	1.773	1.642	
(D) PBE0	7.4931	7.6791	5.7910	333.22	1.914	1.765	1.633	

Reference labels: (B) Burt *et al.* (2006); (W) Winter & Ghose (1979); (A) Aryal *et al.* (2012); (C) Chen *et al.* (2010); (D) Demichelis *et al.* (2010). In each reference column details are given to clearly identify the reference data.

Table S2 Optimized structure parameters of andalusite

Average bond lengths *d* given in Å. *E* is the total free energy.

Reference / Functional	*a* (Å)	*b* (Å)	*c* (Å)	*V* (Å^3)	*d*(Al^{VI}-O)	*d*(Al^{V}-O)	*d*(Si^{IV}-O)	*E* (eV)
Calculations of this study:								
PBE	7.8812	7.9817	5.6129	353.08	1.954	1.853	1.648	-244.866
PBE-D	7.8273	7.9459	5.5965	348.08	1.945	1.846	1.644	-248.646
PBEsol	7.8010	7.9205	5.5830	344.96	1.936	1.840	1.642	-256.085
PBEsol-D	7.7578	7.8917	5.5701	341.01	1.928	1.833	1.639	-259.137
Experimental single crystal XRD references:								
(B) 0.0001 GPa	7.7930 (3)	7.89734 (17)	5.55583 (14)	341.93 (2)	1.93	1.84	1.63	
(W) 25 °C	7.7980 (7)	7.9031 (10)	5.5566 (5)	342.45 (6)	1.935	1.836	1.631	
Calculation references:								
(D) PBE	7.8757	7.9963	5.6311	354.63	1.9547	1.8554	1.6556	
(D) PBEsol	7.798	7.9343	5.599	346.42	1.9367	1.8414	1.6486	
(D) PBE0	7.7935	7.9098	5.5733	343.57	1.9337	1.8374	1.6386	

Reference labels: (B) Burt *et al.* (2006); (W) Winter & Ghose (1979); (D) Demichelis *et al.* (2010). In each reference column details are given to clearly identify the reference data.

Table S3 Optimized lattice parameters of kyanite

E is the total free energy.

Reference / Functional	*a* (Å)	*b* (Å)	*c* (Å)	*V* (Å^3)	*α* (°)	*β* (°)	*γ* (°)	*E* (eV)
Calculations of this study:								
PBE	7.2035	7.9341	5.6355	303.32	89.90	101.13	106.01	-244.24
PBE-D	7.1695	7.8956	5.6044	298.82	89.96	101.10	105.98	-248.86
PBEsol	7.1438	7.8785	5.5877	296.16	90.00	101.11	106.01	-256.12
PBEsol-D	7.1169	7.8471	5.5636	292.65	90.05	101.08	105.99	-259.86
Experimental single crystal XRD references:								
(Co) 0.001 kbar	7.124 (2)	7.856 (2)	5.577 (2)	293.3 (3)	89.99 (2)	101.15 (2)	105.95 (2)	
(W) 25 °C	7.1262 (12)	7.8520 (10)	5.5724 (10)	293.60 (9)	89.99 (2)	101.11 (2)	106.03 (1)	
Calculation references:								
(D) PBE	7.2092	7.9502	5.6516	305.00	89.95	101.14	106.02	
(D) PBEsol	7.143	7.8881	5.6012	297.17	90.06	101.12	106.01	
(D) PBE0	7.1279	7.8635	5.5868	294.86	89.99	101.14	106.01	

Reference labels: (Co) Comodi *et al.* (1997); (W) Winter & Ghose (1979); (D) Demichelis *et al.* (2010). In each reference column details are given to clearly identify the reference data.

Tables S1, S2 and S3 are the result of the benchmark of different functionals listed together with results from the literature for better comparison. The best agreement with experimental lattice parameters is achieved with PBEsol-D.

Table S4 lists selected quantitative and qualitative results of the force field and DFT calculations that were carried out to determine the ideal Al/Si ordering. Note that M40 FFID 86 and M50 FFID 12 represent the Al/Si ordering pattern that in Section 4 is labelled AS3.

Table S4 Characteristics of selected Al/Si permutations

DFT rank refers to the position in the listing of different supercells with the same composition sorted by total energy calculated with DFT (PBE) from lowest to highest. For FFID see Figure 5. For the most stable permutation of each composition (#1) the term E/atom is the total energy of the optimized supercell divided by the number of atoms in the supercell. For higher ranks, E/atom indicates the energy difference ΔE/atom relative to #1. The last five columns state if certain structural elements are present or not. These elements are Si on a *T* site of a tricluster, Si on a *T** site of a tricluster, Si-Si dicluster, Al-Al dicluster and strongly overbonded oxygen as described in the text, respectively.

	FFID	DFT rank	E/atom [meV]	(Al_2SiO_7)	($Al_2Si^*O_7$)	(Si_2O_7)	(Al_2O_7)	SOBO
M0	1	#1	–7639.2	-	-	no	no	no
	2	#2	+29.7	-	-	no	no	yes
	3	#3	+33.7	-	-	no	yes	no

Table S4 continued

M25	2	#1	–7601.0	no	no	yes	no	no
	1	#2	+0.3	no	no	yes	no	no
	13	#3	+2.3	yes	no	no	no	no
	6	#4	+2.5	yes	no	no	no	no
	9		+9.2	no	no	yes	yes	no
M33	1	#1	–7587.4	no	no	yes	no	no
	2	#2	+16.5	no	no	yes	no	no
	6	#3	+19.4	no	yes	no	yes	no
	11	#10	+47.8	no	no	no	yes	yes
M40	3	#1	–7576.4	yes	no	yes	no	no
	8	#2	+1.2	no	yes	yes	no	no
	2	#3	+1.3	yes	no	yes	no	no
	1	#4	+3.0	no	no	yes	no	no
	12	#5	+3.5	yes	no	yes	no	no
	86		+11.5	yes	no	yes	no	no
	4022		+25.3	no	yes	yes	no	no
M50	3	#1	–7559.3	yes	no	no	no	no
	6	#2	+2.7	no	yes	no	no	no
	2	#3	+3.3	yes	no	no	no	no
	1	#4	+7.5	no	no	yes	no	no
	12	#11	+26.1	yes	no	no	no	no
	20	#15	+32.4	*	*	no	yes	yes
	14	#18	+34.0	no	yes	no	no	no
	FFID	DFT rank	E/atom [meV]	(Al_2SiO_7)	($Al_2Si^*O_7$)	(Si_2O_7)	(Al_2O_7)	SOBO

* In M50 #15 ($AlSi_2O_7$) triclusters with two Si are present, one of which occupies a *T** site.

S2. Graphical representation of selected superstructures

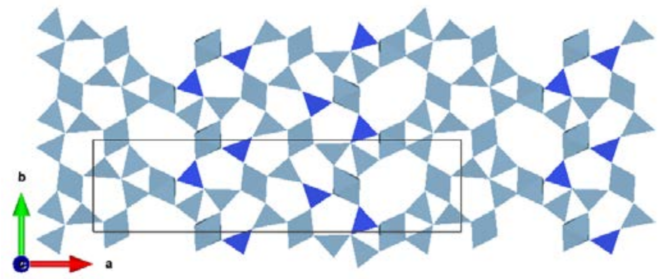

Figure S1 Model of geometrically optimized (PBEsol-D) supercell M50 (AS3)

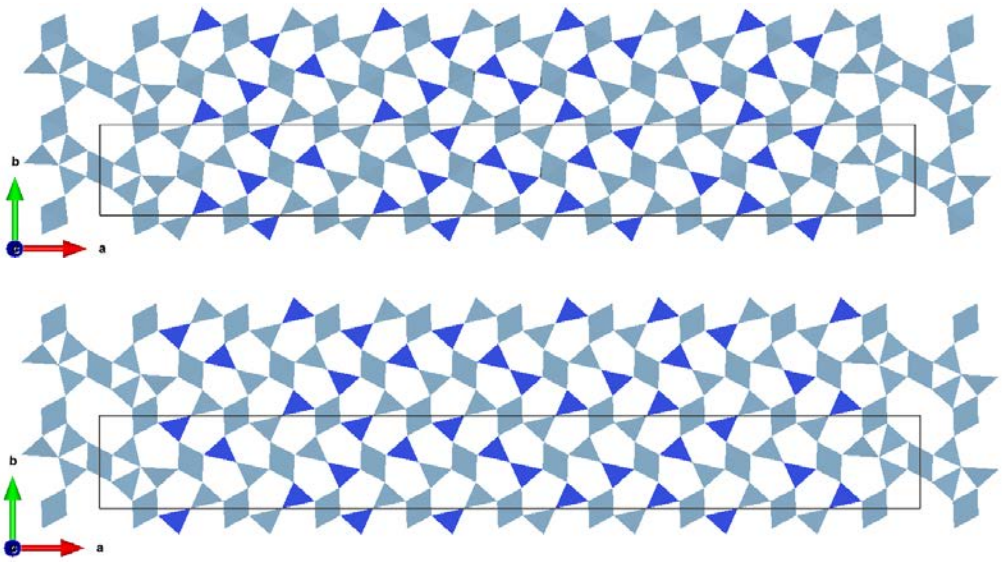

Figure S2 Models of geometrically optimized supercell M11 (top: AS1, bottom: AS2)

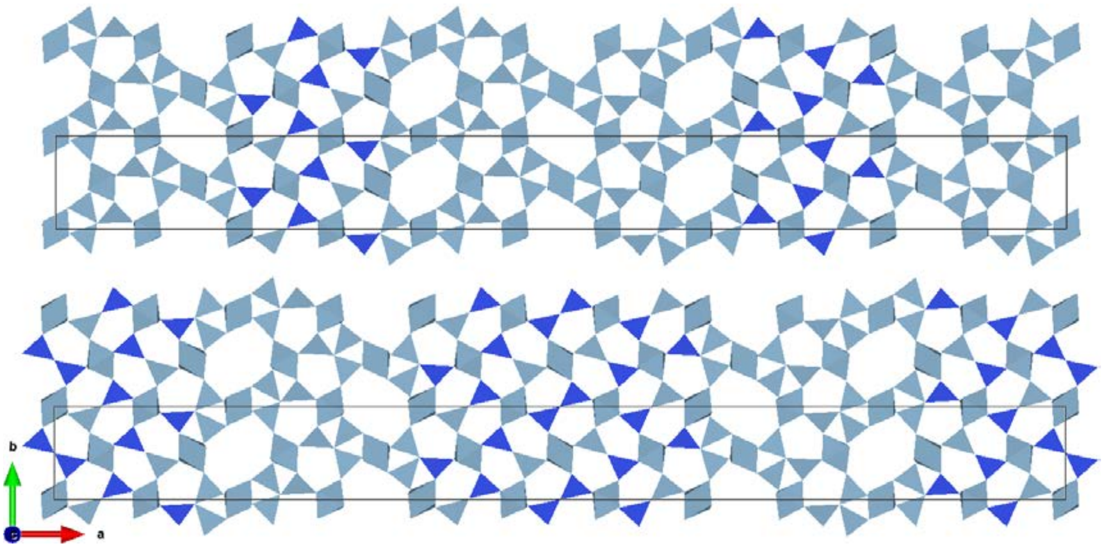

Figure S3 Two subsequent layers of the geometrically optimized (PBEsol-D) supercell M45 (AS1). Note the different number of vacancies per vacancy block.

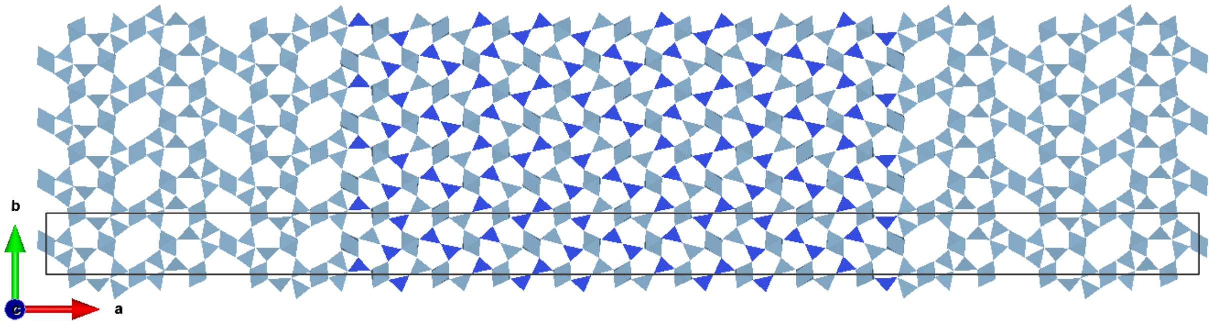

Figure S4 Geometrically optimised (PBE) 19 × 1 × 2 superstructure of M37 (x = 7/19, 594 atoms, *Pnnm*). This calculation shows that larger superstructures can also be analysed with the presented method. Some structures, including this one, were also relaxed with monoclinic symmetry to check if the unit cell angles would change. In all cases the orthorhombic metric was maintained. This structure is not referenced in the main article because the computational cost is very high.

S3. Al/Si ordering M20 (AS2) and M25 (AS2) in superspace

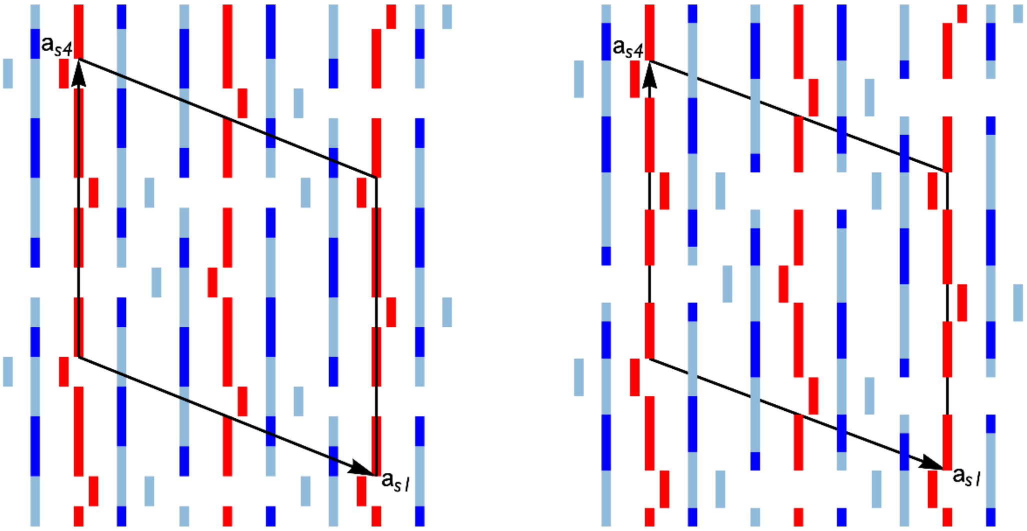

Figure S5 Projections of superspace models of M20 (AS2, left) and M25 (AS2, right)
