# Supplementary figures and images for: Characterizing modulated structures with first-principles calculations: a unified superspace scheme of ordering in mullite
Source: Acta Crystallogr A Found Adv. 2019 Feb 12;75(Pt 2):260–72. doi: 10.1107/S2053273319000846 (PMC6396396; doi:10.1107/S2053273319000846)

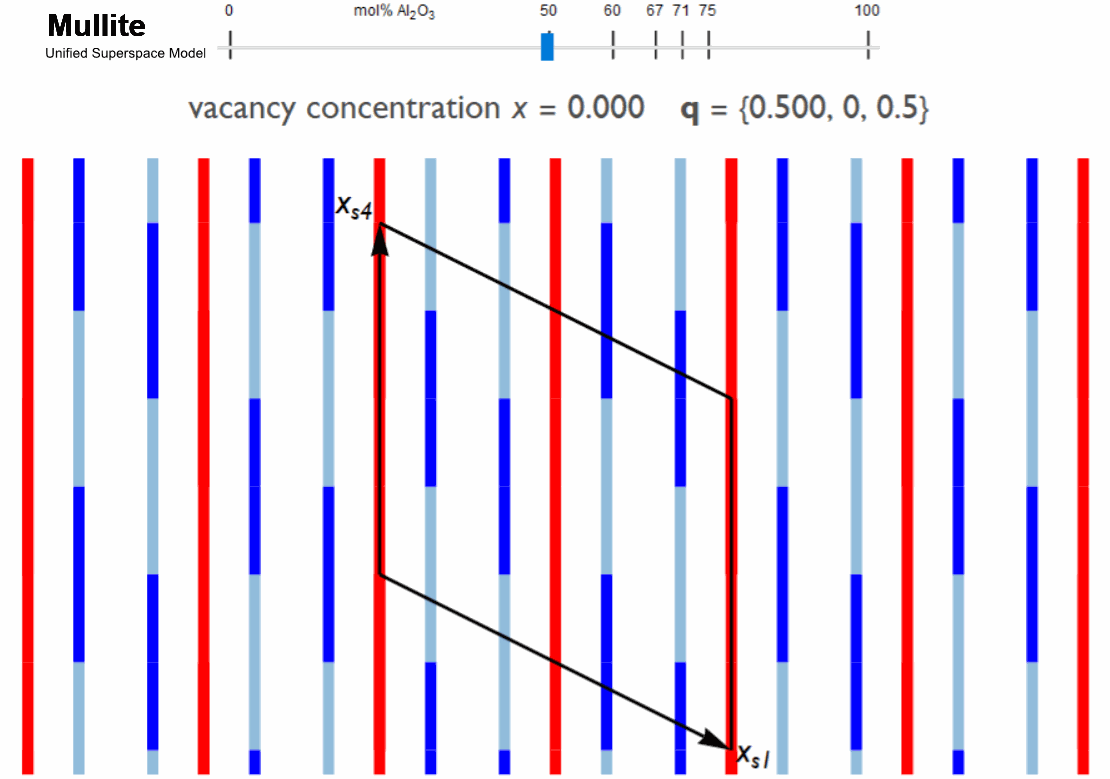

Supplement: Supplementary file 2 [file a-75-00260-sup2.gif]
